# Archipelago-Wide Island Restoration in the Galápagos Islands: Reducing Costs of Invasive Mammal Eradication Programs and Reinvasion Risk

**DOI:** 10.1371/journal.pone.0018835

**Published:** 2011-05-11

**Authors:** Victor Carrion, C. Josh Donlan, Karl J. Campbell, Christian Lavoie, Felipe Cruz

**Affiliations:** 1 Galápagos National Park Service, Galápagos, Ecuador; 2 Advanced Conservation Strategies, Midway, Utah, United States of America; 3 Department of Ecology and Evolutionary Biology, Cornell University, Ithaca, New York, United States of America; 4 Charles Darwin Foundation, Quito, Ecuador; 5 School of Integrative Systems, University of Queensland, Gatton, Queensland, Australia; 6 Island Conservation, Santa Cruz, California, United States of America; 7 United Nations Development Program, Quito, Ecuador; Smithsonian's National Zoological Park, United States of America

## Abstract

Invasive alien mammals are the major driver of biodiversity loss and ecosystem degradation on islands. Over the past three decades, invasive mammal eradication from islands has become one of society's most powerful tools for preventing extinction of insular endemics and restoring insular ecosystems. As practitioners tackle larger islands for restoration, three factors will heavily influence success and outcomes: the degree of local support, the ability to mitigate for non-target impacts, and the ability to eradicate non-native species more cost-effectively. Investments in removing invasive species, however, must be weighed against the risk of reintroduction. One way to reduce reintroduction risks is to eradicate the target invasive species from an entire archipelago, and thus eliminate readily available sources. We illustrate the costs and benefits of this approach with the efforts to remove invasive goats from the Galápagos Islands. Project Isabela, the world's largest island restoration effort to date, removed >140,000 goats from >500,000 ha for a cost of US$10.5 million. Leveraging the capacity built during Project Isabela, and given that goat reintroductions have been common over the past decade, we implemented an archipelago-wide goat eradication strategy. Feral goats remain on three islands in the archipelago, and removal efforts are underway. Efforts on the Galápagos Islands demonstrate that for some species, island size is no longer the limiting factor with respect to eradication. Rather, bureaucratic processes, financing, political will, and stakeholder approval appear to be the new challenges. Eradication efforts have delivered a suite of biodiversity benefits that are in the process of revealing themselves. The costs of rectifying intentional reintroductions are high in terms of financial and human resources. Reducing the archipelago-wide goat density to low levels is a technical approach to reducing reintroduction risk in the short-term, and is being complemented with a longer-term social approach focused on education and governance.

## Introduction

Islands make up a small percentage of the Earth's total area, yet they harbor a large percentage of biodiversity including many threatened and endangered species [1]. Invasive alien mammals are overwhelmingly the major driver of biodiversity loss and ecosystem degradation on islands. Non-native predators, such as rats (*Rattus* spp.) and cats (*Felis silvestris catus*), have decimated endemic vertebrate populations and extirpated seabird colonies on islands around the globe [2], [3], [4]. Non-native herbivores such as goats (*Capra hircus*) have caused wholesale changes to insular plant communities, as well as secondary impacts via habitat degradation [5], [6]. Over the past three decades, however, invasive mammal eradication from islands has become one of society's most powerful tools for preventing extinction of insular endemics and restoring insular ecosystems. [7], [8]. There have been over 780 successful invasive alien vertebrate eradications from islands [9]. Invasive mammals are now being removed from larger islands at a faster rate than ever before [10].

**Figure 1:**
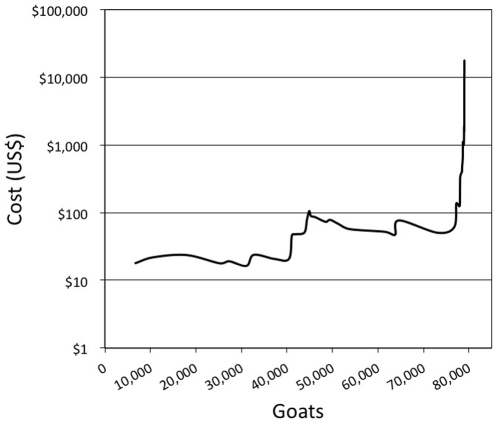
The marginal cost curve of removing goats from Santiago Island, Galápagos (2001–2006). The majority of the 79,569 goats removed cost between US$10–100 per goat to remove. The final goats, however, cost over $10,000 per goat. Technologies and tools targeted at cost-effectively removing the final animals of an eradication campaign could deliver in substantial savings to island restoration programs.

As conservation practitioners tackle larger islands for restoration, three factors will heavily influence success and outcomes: the degree of local support, the ability to mitigate for non-target impacts, and the ability to eradicate non-native species more cost-effectively [10]. The latter is particularly relevant with respect to removing the last animals toward the end of some eradication campaigns. For example, 79,579 goats were removed from Santiago Island in the Galápagos over 4.5 years for a cost of US$6.1 million. The last 1,000 goats cost $2 million to remove over 1.5 years [11]. Decreasing the marginal cost of removing animals from islands is an effective way to increase the environmental return on investment of island restoration programs (Figure 1).

Investments in removing non-native species from islands, however, must be weighed against the risk of reinvasion. If there is substantial risk of reintroduction following a successful eradication, investment in an eradication campaign is not justified. Mitigation strategies for reintroduction risk include biosecurity programs and education [12]. From an archipelago perspective, an effective means to reduce reintroduction risk is to eradicate the target invasive species from the entire archipelago and thus eliminate readily available sources. This approach is valid in the Galápagos as goats are unlikely to come from continental sources and goat breeding doesn't occur on farms.

**Figure 2:**
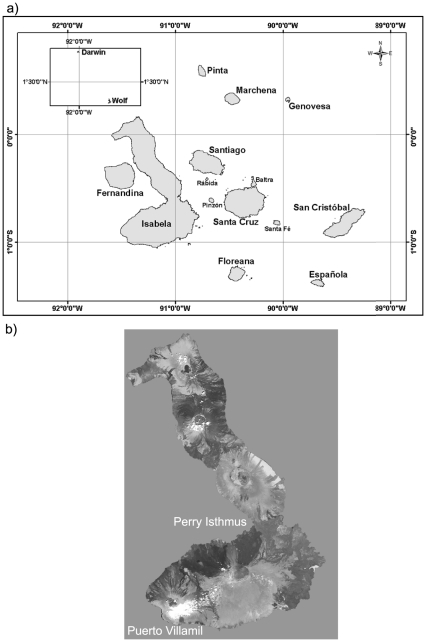
Southern Isabela is, separated from the northern section by a 10+ km-long lava isthmus (Perry Isthmus). A small town, Puerto Villamil, is located at the southern tip of the Island. a) The Galápagos archipelago. b) Isabela Island.

**Figure 3:**
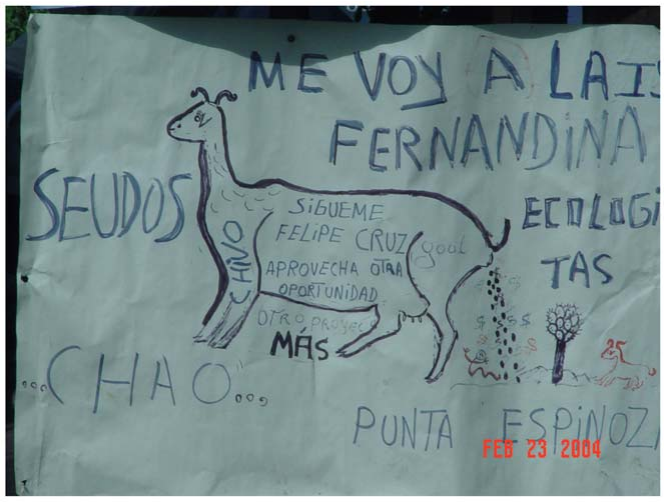
The use of the threat of goat reintroduction to islands in the Galápagos as a political tool. A sign during a 2004 local protest at the Galápagos National Park headquarters, where local fishermen were demonstrating for additional fishing permits. The sign threatens to introduce goats to Fernandina Island, which is the only large island in the archipelago that does not have a history of introduced herbivores.

We illustrate the costs and benefits of this archipelago-wide approach with the efforts to remove invasive goats from the Galápagos Islands (Figure 2), the world's largest island restoration effort to date. The first goat eradication in the Galápagos took place in 1971 on Plaza Sur (12 ha, Table 1). Goats have been eradicated from nine islands since, and populations now remain on only three islands where removal efforts are underway. However, at least nine intentional goat reintroductions have been documented following successful eradication programs (Table 1). A few individuals in the Galápagos Islands have used the reintroduction of goats onto islands as a political tool to influence fishery-permitting processes in the Galápagos Marine Reserve, which is managed by the Galápagos National Park (Figure 3). These reintroductions complicate island restoration efforts, and present a risk to conservation investments in the archipelago that is both difficult and expensive to mitigate. Thus, eradication of goats from the entire Galápagos archipelago is desirable to protect the natural capital of the islands, as well as the massive investment in restoring the islands.

**Table 1:** Island in the Galápagos archipelago where goats have been removed.

Island	Size (ha)	Goats Removed	# Reintroductions	Investment
Marielas Sur	1	5		
Plaza Sur	12	5		
Rábida	499	14	1 (unknown)	
Santa Fe	2,413	3008	1 (unknown)	
Baltra	2,620	64		$9,515
Pinta	5,940	41,683	1 ($110,141)	$83,949*
Española	6,048	3,344		
Marchena	12,996	497	5 ($124,064)	
Floreana	17,253	1,561		$643,705
**San Cristobal**	55,809	7,726		$680,251
Santiago	58,465	79,579	1 ($32,393)	$6,349,326
**Santa Cruz**	98,555	1,481		$281,740
**Isabela**	458,812	62,868		$4,172,035
Total	719,410	201,825	9 ($266,598)	$11,958,282

*Includes eradication efforts from 1999–2003; does not include prior control efforts.

Goats have been removed from over 700,00 ha for a cost of $12 million. Cost data for earlier eradications are not available. Goats have been reintroduced to islands nine times, which has cost more than $266,000 to remove those new populations. Goats remain on the three islands in bold, where removal efforts are underway. All costs are in 2009 US$.

Project Isabela, launched in 1997 with a planning workshop, was a bi-institutional project of the Galápagos National Park and the Charles Darwin Foundation charged to remove pigs (*Sus scrofa*) from Santiago Island, and goats from Pinta, Santiago and northern Isabela Islands, the latter being the largest island in the archipelago. The Santiago Island (58,465 ha) goat eradication campaign, the largest ever attempted in terms of island size and number of animals removed, was mounted as an opportunistic capacity building exercise leading up to goat eradication on northern Isabela Island (458,812 ha). Part of an archipelago-wide invasive species initiative, a goal of the Santiago Island goat eradication campaign was to develop models, techniques, and technologies necessary to cost-effectively eradicate invasive mammals from large islands [11]. Here we report on the details of two goat eradication programs: the efforts to remove goats from northern Isabela Island—the primary objective of Project Isabela, and the post-Project Isabela program to remove goats from the entire archipelago. Details and outcomes of other efforts connected to Project Isabela are reported elsewhere [11], [13], [14], [15], [16], [17]. Leveraging the capacity built during the Santiago and Isabela goat eradications, and given that goat reintroductions have been common over the past decade, we implemented an archipelago-wide goat eradication strategy. That strategy includes aerial hunting across multiple islands to reduce populations to low levels, along with long-term removal and monitoring programs to detect reintroductions and completely eradicate goats from the Galápagos archipelago.

## Results

### Isabela Island Goat Eradication

In 1997, the Galápagos National Park and the Charles Darwin Foundation brought together local staff and 15 international experts in eradication and island restoration. They concluded that goat eradication was possible for northern Isabela at a cost of US$8.5 million over 4 years [18]. The southern section, separated from the northern section by a 10+ km-long lava isthmus, was considered too complex due to a small town located on the southern end and the presence of multiple invasive herbivores and plants (Figure 2). Workshop participants concluded that southern Isabela was best targeted for restoration as a second phase to be planned and implemented following goat eradication on northern Isabela. In 1998, the Global Environment Facility approved an initial funding application for a small project to demonstrate capacity. Subsequently, feral goats were eradicated from Pinta Island in 1999 (Campbell et al. 2004). In 2000, the full-scale Global Environment Facility project was approved (ECU/00/G31 Control of Invasive Species in the Galápagos Archipelago), with the northern Isabela Island goat eradication being the largest component of a holistic approach to invasive alien species management in the Galápagos Islands.

Following the successful removal of goats and pigs from Santiago Island [11], [16], we focused our efforts on removing goats from northern Isabela starting in March 2004. In contrast to Santiago Island, the majority of hunting efforts on Isabela were conducted aerially by helicopter. During the initial phase (April 2004–May 2005), a total of 55,657 goats were killed by aerial hunting. While we concentrated efforts on northern Isabela, the southern part of the island was also hunted. A helicopter accident in June 2004 halted aerial hunting operations for four months. Ground hunting with dogs was limited to seven trips, the majority of which took place in densely vegetated areas. Throughout the entire campaign, only 2,637 goats were killed by ground hunters (Table 2).

**Table 2:** The Santiago and Isabela Island goat eradication campaigns during Project Isabela (2001–2006).

	Santiago	Isabela
Number of goats killed (% total): aerial hunting	12,192 (15%)	55,657 (89%)
Number of goats killed (% total): ground hunting	66,213 (83%)	2,637 (4%)
Number of goats killed (% total): Judas goat operations	1,174 (1%)	4,524 (7%)
Total number of goats killed (cost)	79,579 ($6.4 mm)	62,818 ($4.1 mm)
Duration (months)	64	24
Average $ per hectare	$110	$9
Average $ per goat	$81	$65

Figures do not include Judas goat operations on southern Isabela after March 2006.

Developed and demonstrated during the Santiago Island goat eradication campaign, *Mata Hari* goats, sterilized female Judas goats induced into a long-term estrus, were a critical component of the northern Isabela goat eradication [11], [19], [20]. Judas goats are goats captured, fitted with radio telemetry collars, and released [21]. Being gregarious, Judas goats search out and associate with other goats [22]. Judas goats can be monitored and any associated feral goats killed [21], [22]. By January 2005, goats across all of Isabela Island were at low densities, which precipitated a switch of methods from aerial hunting to Judas goats. Over the next three months over 700 Judas goats were deployed throughout all of Isabela Island, including the southern portion of the island. In contrast to Santiago Island, Judas goat monitoring was conducted exclusively by helicopter. Judas goats were actively monitored on Isabela Island for 465 days. Over that time period, Judas goats were checked 5,470 times; 3,439 feral goats were shot while associated with Judas goats, while 1,085 feral goats were shot during Judas goat operations but were not associated with Judas goats when shot.

The last feral goat on northern Isabela was removed in December 2005. Monitoring operations for Project Isabela ended in March 2006. A total of 62,818 goats were removed from the island over 2 years for a cost of $4.1 million. Feral goats remained in low numbers on southern Isabela. As a future monitoring tool, 266 Judas goats were left on Isabela Island. During Project Isabela operations, donkeys (*Equus asinus*) were also eradicated from northern Isabela, but still persist in small numbers on the southern section of the Island [17].

### Post Project Isabela Operations

Building on the capacity built during Project Isabela, eradication campaigns were mounted on the three inhabited islands were feral goat populations remained: Floreana, San Cristóbal, and Santa Cruz (Figure 2, Table 1). Aerial Judas goat monitoring also continued on southern Isabela; 50 goats and 125 donkeys were removed for a cost $318,000. Goats and donkeys were eradicated from Floreana Island with the use of aerial hunting, ground hunting with dogs, and Judas goats: 1561 goats and 380 donkeys were removed between 2006–2009. A total of 9,207 goats and 498 donkeys were removed from San Cristóbal and Santa Cruz Islands since 2006 using aerial hunting, ground hunting with dogs, and Judas goats. Feral goats currently remain on three islands in the archipelago: a small remnant population on southern Isabela and San Cristóbal Islands and a larger population on Santa Cruz Island. Since 2006, $1.0 million has been spent on removing the remaining goats and donkeys on these islands. Judas goat monitoring is continuing on Floreana and San Cristóbal Islands.

### Goat Reintroductions

Contemporary feral goat introductions and reintroductions are common on the Galápagos archipelago. Fishermen and crew-members on boats travelling in the Marine Reserve commonly introduce goats to islands as a food source, and more recently as a political tool and malicious acts against the Galápagos National Park. There has been at least twelve intentional introductions or reintroductions of feral goats to islands in the Galápagos since 1990 (i.e., on average a goat introduction every 20 months; K. Campbell, unpublished data). In 2008, a goat was even introduced to distant Wolf Island, over 100 km from the inhabited islands. Nine reintroductions have occurred since 2000, which were confirmed as new animals (as opposed to failed eradications) by pelage color or reports by fishermen of the introduction. Managing these reintroductions is a costly business (Table 1). For example, six goats were reintroduced to Santiago Island in 2009, three years after the island was declared goat-free. It cost $32,393 to conduct monitoring since 2008 and remove those goats.

## Discussion

Over the past decade, a suite of innovative invasive mammal eradication programs have significantly increased the pace and capability of island restoration around the globe [23], [24]. Feral pigs were removed from Santa Cruz Island, USA (24,900 ha) in 15 months [25]. Invasive rats were removed from Campbell Island, New Zealand (11,300 ha) [26]. Complicated by the presence of endemic foxes (which were susceptible non-target impacts), feral cats were removed from San Nicolas Island, USA (5,896 ha) in 12 months [27]. On Faure Island, Australia (5,800 ha), cats were eradicated in less than three weeks using both where aerial and ground baiting methods [28]. Project Isabela contributes to the new emerging model of invasive species eradication, which is focused on fast-paced and cost-effective campaigns. Prior to Project Isabela, the largest feral goat eradication took place on San Clemente Island, California (14,800 ha), where 29,000 goats were removed [29]. Project Isabela nearly doubled the total area globally where goats have been removed from islands (567,000 ha) by removing over 140,000 goats from 500,000+ ha in less than 10 years [5].

Over the past decade, removing goats from the Galápagos Islands has become more cost-effective. Those savings stem from a variety of factors. First, the use of helicopters and aerial hunting is more cost-effective at removing non-native herbivores compared to ground-based hunting methods, even in countries where labor is relatively inexpensive (Table 2) [11]. For example, the largely ground-based goat eradication campaign on Santiago Island cost $110 ha^−1^. While a remnant goat population is still present on southern Isabela Island, the current per hectare cost of the aerial-based Isabela campaign is $9 ha^−1^ – a magnitude cheaper. Second, later eradications in the Galápagos were subsidized in the sense of capacity built. The skill level of practitioners was already high, infrastructure existed, the efficiency of techniques had been honed, and the institutional bureaucracy had been navigated; all of these factors resulted in cost savings.

In situations where there will be multiple programs under the same political unit and institutions (i.e., Galápagos National Park), explicitly building long-term capacity within a large project, such as Project Isabela, is strategic. Capitalizing on that built capacity, the Galápagos National Park is now in the final stages of removing feral goats from the entire archipelago. Helicopter contracts for aerial hunting are now part of the Park's annual budget, along with Judas goat operations. Due to the successes of Project Isabela, the Galápagos National Park now has access to the capacity, financing, and political capital to engage in additional biodiversity conservation programs, including the removal of invasive rats from islands and protecting their previous investments in goat eradication. While costs are not available for earlier goat eradication campaigns, at least $12 million has been invested in removing goats from islands in the Galápagos (Table 1).

The reintroduction of goats to islands after they have been eradicated is a real and substantial risk to massive restoration investments made by the Galápagos National Park and international community. Goat reintroductions over the past decade have been sourced from Santa Cruz and San Cristóbal Islands, where until recently, goats were abundant. Those source populations have been made inaccessible by the initial reduction of goat populations on those two islands with ground and aerial hunting. The costs of rectifying intentional reintroductions are quite high in terms of financial and human resources. Reducing the archipelago-wide goat density to low levels is a technical approach to reducing reintroduction risk, and is being complemented with a longer-term social approach focused on education and governance [30]. Both approaches are important, particularly in socio-political settings that are volatile like the Galápagos Islands, where unforeseen events are common (e.g., future fishing regulations result in malicious behavior by a few individuals resulting in intentional goat reintroductions to restored islands). The Galápagos National Park manages 97% of the archipelago, with the remaining land made up of residential land and farmland. Goats are not bred on farms as livestock in the Galápagos archipelago; however, some goats are captured and maintained by locals while hunting. The likelihood of goat reintroduction from the mainland is unlikely, since it is ∼1000 km away. By reducing goats to low densities archipelago-wide and then moving forward on eliminating goats completely, the risk of goat reintroductions is drastically reduced. To date, this archipelago-wide approach appears to be working.

As conservation practitioners tackle larger and more biologically complex islands for restoration, the biodiversity benefits must clearly outweigh the costs and risk of failure. The goat eradication efforts on the Galápagos Islands have delivered a suite of biodiversity benefits that are in the process of revealing themselves and being documented. The endangered Galápagos rail (*Laterallus spilonotus*) has made a spectacular recovery on multiple islands following vegetation recovery, including on Floreana Island where they had not been documented since the late 1980s [6], [31]. The native plant communities on Pinta, Santiago, Isabela and Floreana Islands are recovering. Populations of eight endemic plant species listed by the International Union for the Conservation of Nature have increased in both number of populations and individuals, including the endangered *Scalesia atractyloides* that was feared extinct [32]. The fast recovery of this endemic tree following goat eradication on Santiago Island has led to a proposal to downgrade its endangered status [33]. Since invasive herbivore eradication is but one step in the restoration process, however, other conservation challenges have presented themselves post-eradications. Despite concurrent invasive plant control during Project Isabela [11], blackberry (*Rubus niveus*) has now become more common in the highlands of Santiago, likely due to the release of herbivore pressure and the constant dispersal of seeds by native birds. Systematic control and containment efforts are now being implemented and investigations to identify bio-control agents is planned for blackberry by the Galápagos National Park.

The efforts on the Galápagos Islands have demonstrated that for invasive mammalian herbivores, island size is no longer the limiting factor with respect to eradication. Rather, costs, financing, and stakeholder approval appear to be the new challenges. For example, the removal of three invasive mammals from the remote Macquarie Island, Australia (12,780 ha) is budgeted at $AUS24.7 million [34]. Those massive investments in political and financial capital must be protected, including minimizing the potential for species reintroductions following eradication. Our archipelago-wide goat eradication approach has been successful in removing any readily available sources of goats for potential reintroduction by either eradication or reducing remaining populations to low densities. The three remaining goat populations are currently in the process of being removed. Feral goats were already present in the Galápagos when Charles Darwin arrived in 1835. One hundred and seventy-six years later, the archipelago is quickly on its way to becoming goat-free. At a cost that will likely be less than $20 per hectare, invasive mammal eradication from islands is not only one of society's most powerful tools for preventing extinction and restoring ecosystems—but also one of the most cost-effective.

## Methods

### Aerial Hunting

The aerial component consisted of 2 helicopters (MD500D/E, McDonnell Douglas, AZ) with pilots and shooters highly experienced in aerial hunting. We used two, sometimes three, shooters per helicopter. Aerial shooters used semi-automatic 12 gauge shotguns (M1 Super 90, Benelli, Urbino, Italy) and semi-automatic .223 caliber AR15 rifles (JP15, JP Enterprises, MN). Shooters tracked number of animals shot with manual counters, and the pilot recorded the location and relative numbers shot with a GPS. We partitioned islands into blocks for aerial hunting. We determined block size primarily by openness of vegetation, relative goat density, and helicopter flight times, which were limited to 2 hours. We hunted the blocks until the kill rate reached <5 animals hour^−1^. The aim was to remove as many animals as possible in the first sweep. Once two or three blocks reached the target kill rate, they were combined into larger blocks. Minimizing escapes while aerial hunting was the priority. Goats quickly became educated and wary, often hiding in bushes, caves, or lava tunnels. Aerial shooters were often dropped off to hunt goats that were in refuges. Toward the end of the aerial hunting campaign and prior to releasing Judas goats (see below), islands were hunted several times consecutively.

### Ground Hunting

Ground hunters with specialized hunting dogs were used in select areas and times on Isabela Island, along with operations on Santa Cruz, Floreana, and San Cristóbal Islands. We deployed ground hunters in areas with dense vegetation, where aerial hunting often proved inefficient. Ground hunting teams varied in size from 10–28 and consisted of locals, all of which were highly skilled due to extensive training and experience during the pig and goat eradications on Santiago Island. We trained hunters in all facets of hunting and field skills, including dog handling, ethics, GPS, radios, rifles, first aid, and telemetry. Ground hunters used .223 caliber rifles (Ruger, Southport, CT) and 55-grain pointed-soft-point ammunition (Winchester, East Alton, IL). Every hunter carried a GPS and recorded their daily movements. Hunters also recorded a variety of spatial and non-spatial data, including kills, escapes, sex, location, and area traveled [14], [15]. Hunters collected tails to confirm reported kills, except when a hunter shot >80 animals/day, and tail collection decreased hunting efficiency.

### Judas Goats and Monitoring

We live-captured goats from Isabela Island by mustering them into corrals by helicopter, or capturing them directly with the aid of a helicopter. Each Judas goat was ear-tagged with a unique number, fitted with radio telemetry collars with a unique frequency, and quarantined if being transported to another island. We then sterilized female and male Judas goats, and terminated any pregnancies [20]. In conjunction with a field experiment on Santiago Island to assess the efficacy of different types of Judas goats, we used a combination of three type of Judas goats: males, females, and females with hormone implants; the latter coined *Mata Hari* goats [11], [19]. The results of those field experiments are and will be published elsewhere [11], [14], [35]. Judas goats were deployed at 2.25 km equidistant spacing in vegetated zones and 3 km spacing in areas sparsely vegetated and dominated by lava. Between March 2005—March 2006, some 700 Judas goats were deployed across northern and southern Isabela Island. Judas goats were monitored by helicopter. We captured Judas goats associated with other Judas goats and re-deployed them in vacant areas; we constantly updated those areas with maps containing the last monitored position for each Judas goat. We collected DNA samples from Isabela Island and remaining goat populations in the archipelago. If goats are found in the future, it may be possible to determine whether they were introduced from another island or local goats that evaded eradication efforts [36].

### Economics of Eradication

We tracked all costs and effort associated with the eradication campaigns. We calculated cost per effort for each activity (e.g., $ dog hour^−1^, $ helicopter hour^−1^), incorporating salary, administration (including institutional overhead), management, and logistical costs. We assigned percentages of time or resource use of each cost of the 4 principal methods: helicopters, hunters, hunting dogs, and Judas goats. We converted all costs to 2009 US$ unless noted otherwise. Some existing infrastructure was already in place on-island (i.e., trails, huts) and was not included in our reported costs. Those costs comprised a small fraction of overall eradication campaign expenditures.

### Ethics Statement

Project Isabela did not require ethics approval. However, aspects of the Judas goat program were conducted with the University of Queensland's Animal Ethics Committee approval (#NRSM/388/00/UQ/CDF/GNPS).
